# Photosensor-Based Latency Measurement System for Head-Mounted Displays

**DOI:** 10.3390/s17051112

**Published:** 2017-05-15

**Authors:** Min-Woo Seo, Song-Woo Choi, Sang-Lyn Lee, Eui-Yeol Oh, Jong-Sang Baek, Suk-Ju Kang

**Affiliations:** 1 Department of Electronic Engineering, Sogang University, Seoul 04107, Korea; yoynok08@gmail.com (M.-W.S.); songwoo602@gmail.com (S.-W.C.); 2 LG Display, Paju 10845, Korea; sanglyn@lgdisplay.com (S.-L.L.); eyoh@lgdisplay.com (E.-Y.O.); bjs@lgdisplay.com (J.-S.B.)

**Keywords:** head-mounted display, virtual reality, latency measurement system

## Abstract

In this paper, a photosensor-based latency measurement system for head-mounted displays (HMDs) is proposed. The motion-to-photon latency is the greatest reason for motion sickness and dizziness felt by users when wearing an HMD system. Therefore, a measurement system is required to accurately measure and analyze the latency to reduce these problems. The existing measurement system does not consider the actual physical movement in humans, and its accuracy is also very low. However, the proposed system considers the physical head movement and is highly accurate. Specifically, it consists of a head position model-based rotary platform, pixel luminance change detector, and signal analysis and calculation modules. Using these modules, the proposed system can exactly measure the latency, which is the time difference between the physical movement for a user and the luminance change of an output image. In the experiment using a commercial HMD, the latency was measured to be up to 47.05 ms. In addition, the measured latency increased up to 381.17 ms when increasing the rendering workload in the HMD.

## 1. Introduction

**Figure 1:**
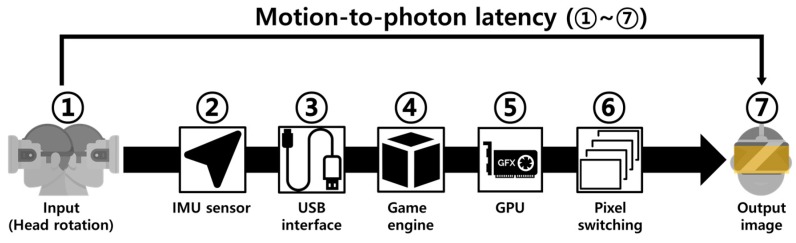
Overall process and motion-to-photon latency of the image rendering.

Nowadays, movies and game contents using virtual reality (VR) have taken a center stage because they provide greater immersion and realism. Thus, the VR market is expected to expand rapidly [1]. Especially, the VR environment using a head-mounted display (HMD) is currently in the spotlight as a new growth market because of its reasonable price and accessibility, compared with any other VR equipment [2]. However, HMD devices may have several problems, such as a screen-door effect [3] caused by the low spatial resolution, a frame rate drop caused by the low computing performance, and a blurring artifact caused by the low temporal resolution. Among the problems, the motion-to-photon latency is the most significant one [4] because it results in motion sickness and dizziness caused by the inconsistency of human perception. Specifically, it refers to the difference between the starting time point of the head motion for a new orientation and the time point when generating an image on the display of an HMD system [5]. Figure 1 shows the overall process and motion-to-photon latency of the image rendering in an HMD system. First, the physical head movement occurs, and the head position is measured using an inertial measurement unit (IMU) sensor. Then, an HMD device transmits the measurement data to a PC via a USB connection. The PC generates the changed image in the virtual space based on the measured physical position using the graphics processing unit (GPU). Eventually, a new image is outputted to the display of the HMD system. In this case, each module has a latency, and the total summation of the latencies in the whole process is called the motion-to-photon latency [6,7].

Reducing the latency requires an accurate measurement system that can consider the human physical movement [8]. The following section explains the conventional approaches and their problems.

## 2. Previous Work

**Figure 2:**
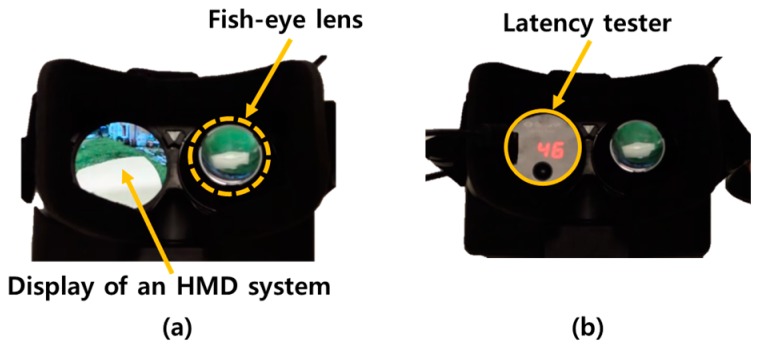
Conventional latency measurement device: Oculus latency tester (**a**) before installing latency tester and (**b**) after installing latency tester.

**Figure 3:**
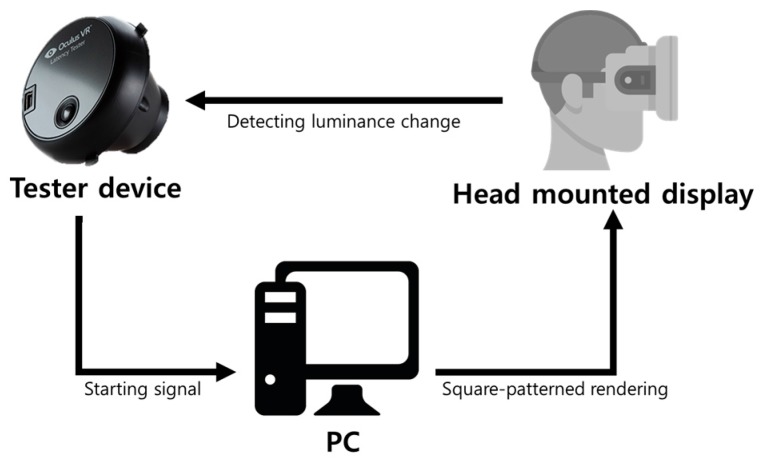
Measurement procedure of the Oculus latency tester.

As a conventional and commercial measurement device, the Oculus latency tester [9] is the only one available. Figure 2 shows the tester installed for the latency measurement, and Figure 3 shows its measurement procedure. The tester sends a starting signal to a PC via a USB connection by pushing a button. After receiving that signal, the PC generates a square-patterned image and combines it with the current output image. Then, it outputs this image to the display of the HMD system. As shown in Figure 2, a photosensor of the tester mounted on the left side of the fish lens in the HMD continuously measures whether the luminance value for the particular color on a specific location of the display is higher than the threshold value, which is predetermined by the Oculus tester. If it is satisfied, the difference, which is the motion-to-photon latency, between the starting time and the measured time in the display of the HMD system is calculated.

However, the existing measurement system has three problems. First, it does not consider a physical head movement and simply measures the luminance change between the input and the output signals in the screen. The second one is compatibility. The Oculus latency tester is exclusively dependent on the Oculus Rift DK1 hardware. Thus, it cannot be used for other HMD devices, nor can it be used as a reference measurement system for measuring the latency. The third one is the low accuracy. The conventional method starts to measure the latency by pressing the button on top of the tester device. Using the signal activated from the tester device, the PC renders the specific patterns and sends them to the display of the HMD system. Therefore, it does not consider the physical movement of the HMD system (it may have several milliseconds of latency). In addition, it is impossible to measure the latency change by changing the workload of the rendered image.

In a previous research [10], a new measurement system that can solve the abovementioned problems was proposed. However, it cannot ensure the accuracy owing to the use of a low-performance servo motor and because the output image uses the mirroring mode, which is a method that outputs an image to a monitor rather than to the display of an HMD system. In addition, it cannot precisely model the head movement. In other methods, a pendulum-based system [11] can measure the latency using a camera, but the pendulum motion cannot model the head movement, and it is not appropriate as a measurement system for recent HMDs with a small latency change due to the low accuracy. In a similar way, there is a conventional method calculating the latency based on the phase changes, which are measured in the virtual space and real space. This method has high precision by using the photosensor and rotary potentiometer, but it cannot provide the accurate latency because it contains many approximations in the motion generation and mathematical modeling [12]. The end-to-end measuring instrument [13] cannot consider the head motion because only one axis can be considered. In addition, HMDs use small OLED panels and, hence, the measurement equipment must be attached in front of the panel to detect the pixel luminance change. However, this system uses the monitor instead of the panel of the HMD. The method of [7] can measure a latency of the HMD system with the high sampling rate. However, this method cannot be used in various types of commercially available HMDs.

In this paper, a novel latency measurement system with high accuracy for solving these problems is proposed. The proposed system can generate an accurate movement of the HMD and measure the movement using high-accuracy encoders and motors. Then, a photodetector system detects the luminance of the changed image for multiple movement directions and calculates the time difference between two events. Therefore, it has high accuracy and reliability and can accurately measure the motion-to-photon latency, which is a critical performance indicator of an HMD system. In addition, the proposed method is easy to apply to various kinds of HMD systems because it is developed considering the characteristics of common HMDs. Table 1 shows the summary for considerations of the conventional and proposed latency measurement methods. In addition, the rightmost column shows where each method can measure the latency in the entire HMD system with reference to Figure 1.

In summary, this paper offers the following contributions:

- Consideration of the physical head movement
- Compatibility between various HMD devices
- High measurement accuracy
- Easy applicability to various kinds of HMD systems.

## 3. Proposed Measurement System

**Figure 4:**
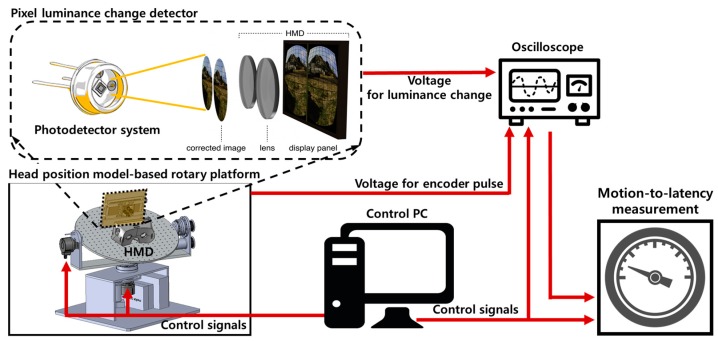
Conceptual architecture of the proposed latency measurement system.

Figure 4 shows a conceptual architecture of the proposed latency measurement system. The proposed system largely consists of a control PC, a head position model-based rotary platform, a pixel luminance change detector, and a digital oscilloscope.

**Figure 5:**
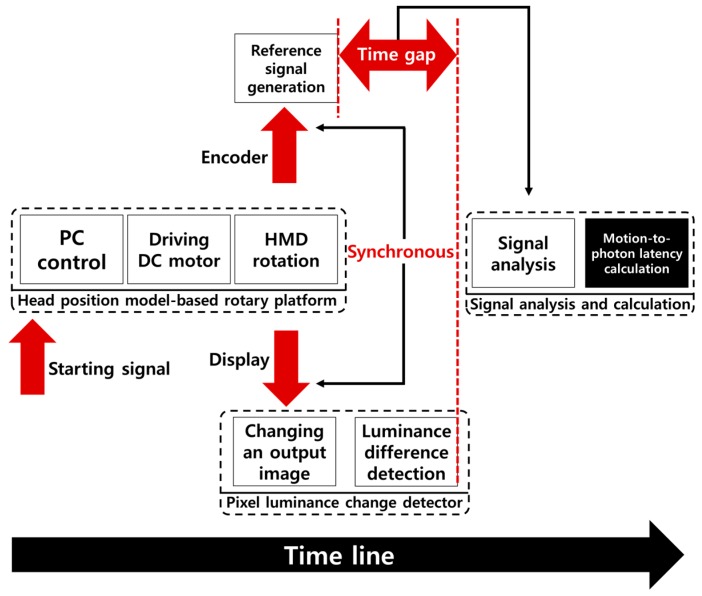
Overall procedure of the proposed system.

The control PC controls each module and analyzes the measured signals, and the rotary platform is a physical device for modeling a head movement and for measuring the movement determined by the high-accuracy encoders and motors. The pixel luminance change detector measures the luminance change in the display of the HMD system and converts it into a voltage value. The oscilloscope measures and displays the voltage values of the measured signals. Figure 5 shows the overall procedure for the proposed system shown in Figure 4. First, the head position model-based rotary platform generates a precise head movement as determined by the control PC. Second, the photosensors of the pixel luminance change detector measure the changes in pixel luminance in the display of the HMD system caused by rotating the platform. Then, the oscilloscope displays the measured voltages for the physical movement of the platform measured by the encoders and the luminance change measured by the photosensors. Finally, the control PC calculates the motion-to-photon latency. The detailed explanation is given in the following subsections.

### 3.1. Head Position Model-Based Rotary Platform

**Figure 6:**
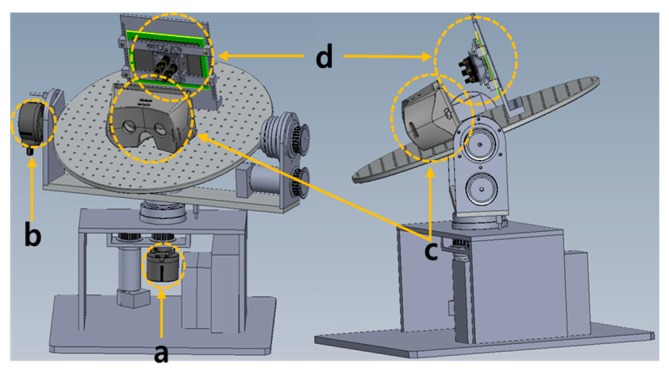
Overall architecture of the photosensor-based latency measurement system: (**a**) a yaw-direction encoder, (**b**) a pitch-direction encoder, (**c**) a HMD system, and (**d**) a plate holding the display of the HMD system.

**Figure 7:**
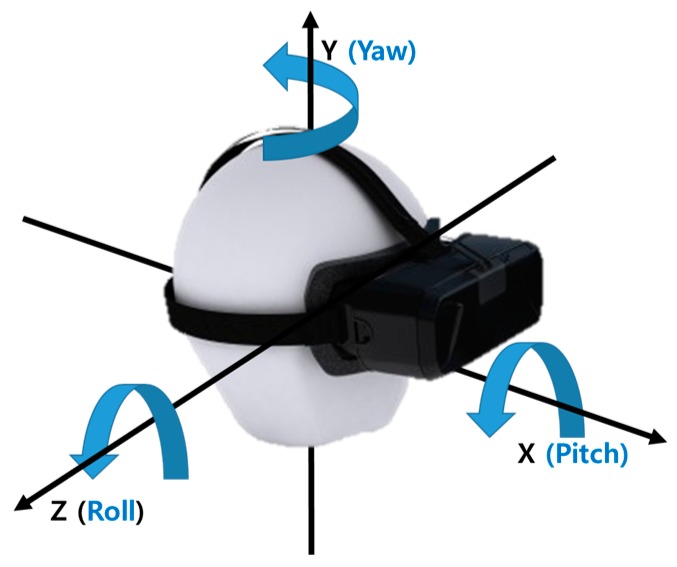
Euler angles and coordinates in the HMD system.

Figure 6 shows the overall architecture of the proposed photosensor-based latency measurement system. The rotary platform fixes the HMD system to the circular top plate and uses two motors and encoders for the rotation and position detection. Specifically, it can rotate in two axes of the yaw and pitch directions for modeling the head movement, as shown in Figure 7.

In this case, the typical range for the head movement is the rotation of the yaw direction, which is up to ±50°, and the rotation of the pitch direction, which is up to ±40°. The maximum angular velocity of the head movement is up to 780°/s in the yaw direction and up to 380°/s in the pitch direction [14]. The proposed system is operated considering these human constraints. It also uses high-performance encoders with high resolution to improve the measurement accuracy and to measure precisely the rotation angles. Specifically, it has an accuracy of 0.018°/step based on an optical incremental-type rotary encoder (the step is a unit slit width of the rotating disk in the encoder). Figure 6a shows the first encoder, which measures the rotation angle of the yaw direction, and Figure 6b shows the second encoder, which measures the rotation angle of the pitch direction. Figure 6c shows the circular top plate where the HMD system is fixed, and Figure 6d shows a plate holding the display of the HMD system, which performs the pixel luminance change detection. The detailed operation of the proposed head position model-based rotary platform is discussed as follows: First, the head movement scenario defined by users is inputted into a control PC, and the proposed platform drives the DC motors to control the rotary platform for performing the head movements. Then, the movements in the pitch and yaw direction are performed, and the HMD system attached to the top plat-form is also moved. At the same time, each axis encoder generates pulses with different phases according to the movement because it prevents the interference between two different movements (the phase difference is 90°). Therefore, the physical movement can be detected accurately.

### 3.2. Pixel Luminance Change Detector

**Figure 8:**
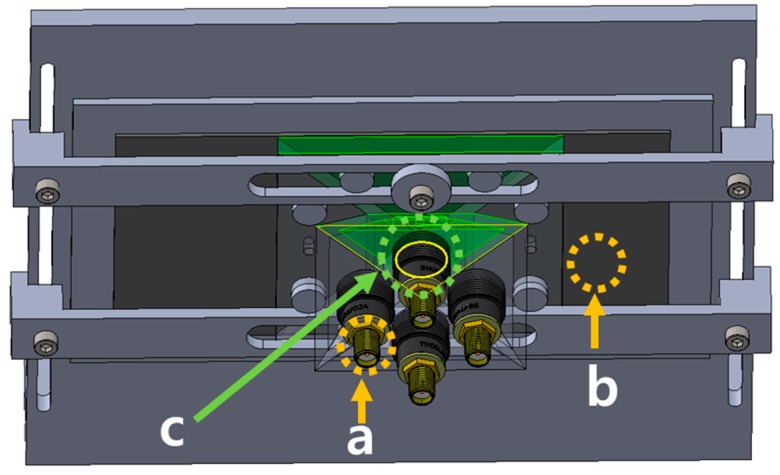
Architecture of the pixel luminance change detector: (**a**) a photosensor, (**b**) an HMD panel, and (**c**) a chamber.

**Figure 9:**
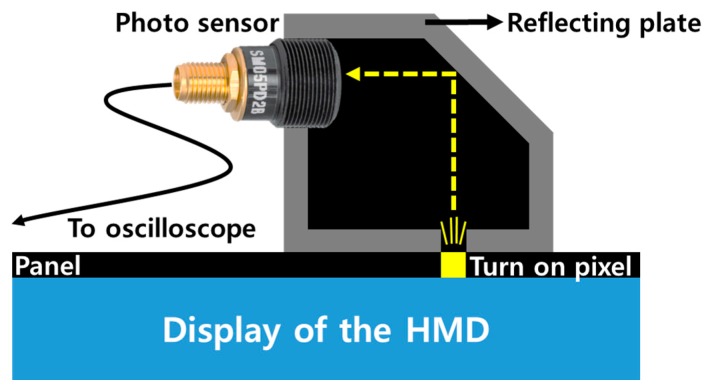
Cross-sectional diagram of an individual pixel luminance change detector.

Figure 8 shows the overall architecture of the pixel luminance change detector used in the proposed system. It is placed on the rotary platform. The upper deck supporter of the detector holds the HMD system’s display panel, which outputs the rendered image. Four separate photosensors are used to recognize the direction, and they are located in front of the HMD panel, as shown in Figure 8a,b shows the HMD panel, which outputs the images, and Figure 8c shows a chamber that connects the photosensors and the display panel. Figure 9 shows a cross-sectional diagram of an individual pixel luminance change detector. It blocks the entrance of the external light between a panel and a photosensor, and it only transfers the light emitted from the panel to the photosensor. To measure the low-level change in pixel luminance in the desired position, a small slit where light could enter from the panel to the chamber to generate a darkroom environment.

**Figure 10:**
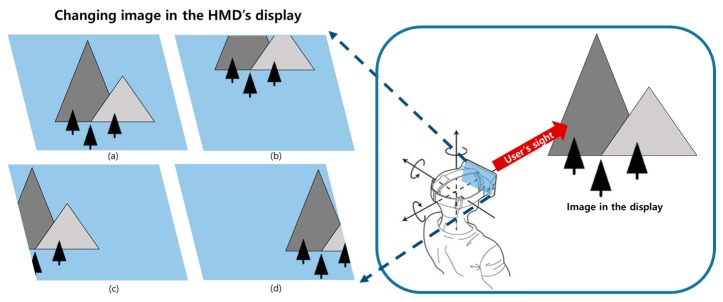
Examples of image changes according to the physical movement of the HMD system: (**a**) an initial image, (**b**) an image generated when a user moves down, (**c**) an image generated when a user moves right, and (**d**) an image generated when a user moves left.

**Figure 11:**
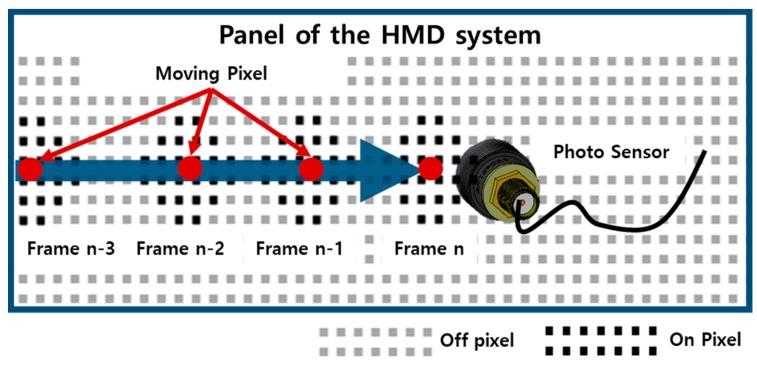
Operation process of the pixel luminance change measurement method.

The operation process of the pixel luminance change detection is as follows. The output image of the HMD panel is changed according to the HMD movement, as shown in Figure 10 [15]. The HMD system outputs an image corresponding to the gaze of the user in the virtual space. For example, if a user looks at the front, the HMD system outputs an image like that shown in Figure 10a. Figure 11 illustrates the concept of the pixel luminance change measurement method in the display. A display consists of multiple pixels, and hence, the image movement is represented by on-off pixels. If an object in the display panel moves toward the sensing position of the photosensor, as shown in Figure 11, the on-off switching of the pixel would be changed from the (*n-3*)-th frame to the (*n*)-th frame. The changed luminance is converted into a voltage in the photosensor, and this voltage is measured using the oscilloscope. This permits the luminance change in the screen to be measured.

**Figure 12:**
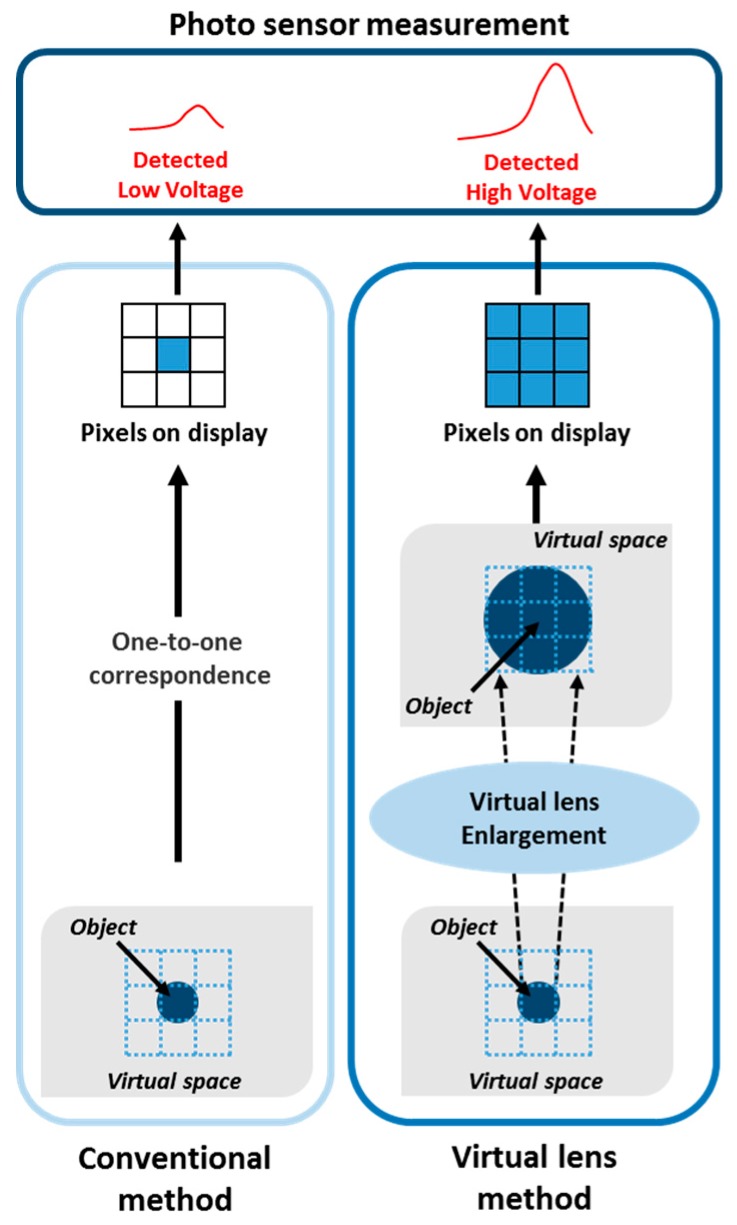
Proposed virtual lens technique for improving the accuracy of the measurement.

In this case, the luminance change in the virtual space should be considered instead of the pixel luminance change in the display panel because a change occurs in the image of the virtual space based on the head movement. In some cases, the luminance change could not be measured because the pixel luminance change is not sufficient enough to be detected by the photosensor. To solve this problem, a virtual lens technique is proposed as shown in Figure 12. This technique enlarges the pixel luminance as if using a magnifying glass, and hence, can measure the low-level luminance change in a pixel. The typical coordinate mapping between a 3D virtual space and a 2D space is as follows:

(1) $$I_{out}^{2D}\left(x,y\right)=I_{out}^{3D}\left(x_{sq},y_{sq}\right),$$

where *I^3D^_out_* and *I^2D^_out_* denote the luminance in the 3D virtual space and 2D mapped space in the display, respectively. *x_sq_* and *y_sq_* denote the horizontal and vertical indexes, respectively, which are sampled and quantized. *x* and *y* denote the horizontal and vertical indices in the 2D space, respectively.

Using (1), the proposed method using the virtual lens is defined as follows:

(2) $$I_{out}^{f}=\;\overset{K}{\underset{x=-K}{\sum }}\overset{K}{\underset{y=-K}{\sum }}I_{out}^{2D}\left(x,y\right),\;K=\lfloor p/2\rfloor$$

where *p* denotes the magnification of the virtual lens and *I^f^_out_* denotes the final output luminance. The proposed system uses *p* = 5, which was experimentally selected. If *p* is higher, the output luminance is also higher, and it is changeable.

### 3.3. Signal Analysis and Calculation

The signal analysis and calculation modules finally compute the motion-to-photon latency using two signals, namely, a pulse from the encoder for the physical movement and a pulse from the photosensor measuring the luminance change. In this case, it is important to separate exactly an original signal from a noise signal by considering the sensitivity of the signal change. The proposed system uses a thresholding technique to remove noise. It is based on the change in measured signals to set the optimal threshold value. Specifically, it measures the number of n signals without changing the signal during a specific period as follows:

(3) $$\begin{array}{l} V_{m}=\frac{1}{N}\overset{N}{\underset{t=1}{\sum }}V_{in}\left(t\right),\\ N_{c}\left(t\right)=V_{in}\left(t\right)-V_{m},\\ N_{e}=\max\left\{\left|N_{c}\left(t\right)\right|\right\},\;1\leq t\leq N, \end{array}$$

where *V_in_* denotes an input signal at time t and N denotes the total number of input signals during a specific period. *V_m_* denotes an average voltage of the input signals with noise. *N_c_* denotes a candidate noise at time *t*, and *N_e_* denotes an estimated noise, which is the largest absolute value among candidate noises. It is set to the threshold value, and the proposed method only selects the voltage beyond this value. Finally, the time difference between two signals, namely, a pulse from the encoder and a pulse from the photosensor measuring the luminance change in the display of the HMD system, is measured. It is defined as follows:

(4) $$\Delta t=\left|t_{photo}-t_{encoder}\right|,$$

where *t_photo_* denotes the time point when the luminance changes in the HMD display, *t_encoder_* denotes the time point when the physical movement of the HMD occurs, and Δ*t* denotes the final motion-to-photon latency.

## 4. Implementation

**Figure 13:**
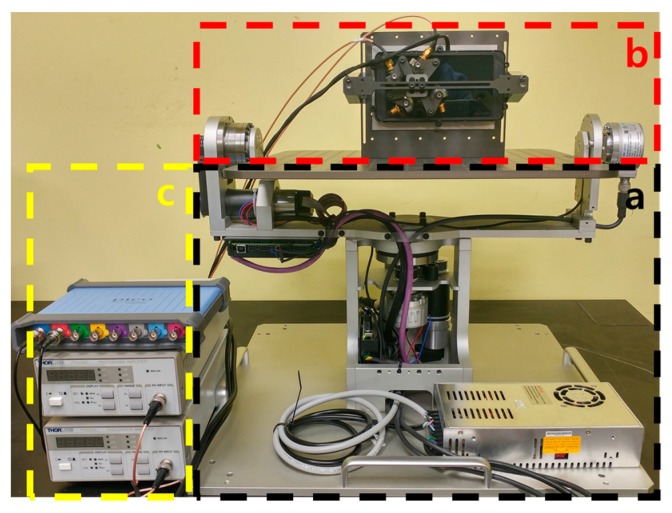
Prototype of the proposed latency measurement system: (**a**) a head position model-based rotary platform, (**b**) a pixel luminance change detector, and (**c**) an oscilloscope and an amplifier.

Figure 13 shows an implementation of the photosensor-based latency measurement system. The rotary platform was designed to make a movement, as shown in Figure 13a, and the detector was placed on this platform to measure the luminance change, as shown in Figure 13b. The oscilloscope and the amplifiers, shown in Figure 13c, were used to calculate and analyze the output signals of each part. Specifically, the Oculus Rift DK2 hardware, which is one of the most popular VR systems, was used as the target HMD. A rotary DC motor (RE40, Maxon, Sachseln, Switzerland) [16] was used to rotate the platform, and a controller (EPOS2 50/5, Maxon, Sachseln, Switzerland) was used to handle the platform [17]. In addition, incremental-type encoders (EIL580, Baumer, Southington, CT, USA) [18] were used to generate pulses based on the movement of the HMD. Its maximum output frequency was 300 kHz and its resolution was 5000 steps/turn (0.018°/step). A photosensor (SM05PD2B, Thorlabs, Newton, MA, USA) was used to measure the luminance change [10], and its spectral range was from 200 nm to 1000 nm. The PC-based oscilloscope was a PicoScope 4824 oscilloscope (Pico technology, St. Neots, UK) [19]. For rendering the virtual space and analyzing the signals, a PC with an Intel i7-6700k 4.4-GHz CPU and an NVIDIA GeForce GTX 1080 GPU was used. In addition, the time-warp technique was not used for generating VR patterns used to measure the actual motion-to-photon latency of the HMD.

## 5. Experiment Result

Two different experiments for the latency measurement were performed using the proposed system. First, the motion-to-photon latency for the commercial HMD was measured. Second, this latency was evaluated by changing the graphic rendering workload for the HMD system.

**Figure 14:**
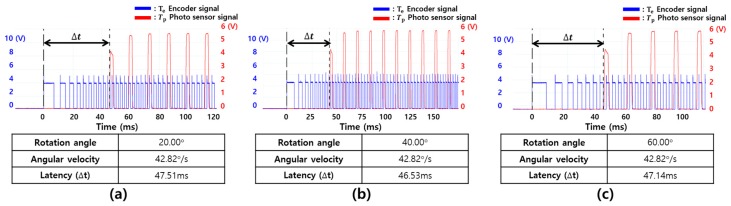
Latency measurement results when the yaw rotation angle was changed: (**a**) 20°, (**b**) 40°, and (**c**) 60°.

**Figure 15:**
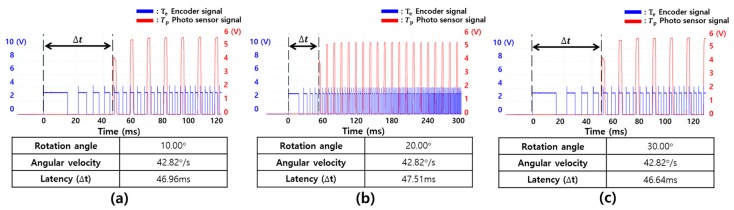
Latency measurement results when the pitch rotation angle was changed: (**a**) 10°, (**b**) 20°, and (**c**) 30°.

First, motion-to-photon latencies were measured by rotating in the yaw and pitch directions. Table 2 shows the statistical results for the experiment repeated 20 times to measure the rotation angle of the yaw direction. The measured average latencies were almost constant, and the standard deviations were also low. This means that the proposed instrument can accurately measure latency without any deviation. Figure 14 shows the motion-to-photon latency for a specific individual experiment. The blue pulse was generated by the encoder with high resolution, which was used for the physical movement. The red pulse was generated by changing the luminance of the display in the photosensor. The rotation angles were 20°, 40°, and 60°, and the results were 44.61, 46.83, and 46.46 ms, respectively. Each of the standard deviation was also calculated up to 1.45 ms. The latency was measured accurately regardless of the rotation angle in the yaw direction. Next, Table 3 shows the experimental statistical results for the rotation of the pitch direction. In this case, the rotation angles were 10°, 20°, and 30°, and the results were 46.48, 46.79, and 47.05 ms, respectively. The standard deviation was up to 1.09 ms. Figure 15 shows the latency in the pitch direction for a specific individual experiment. These results showed that the proposed measurement system could measure the latency precisely and also showed the reliability of the measurement because of the almost similar results.

**Figure 16:**
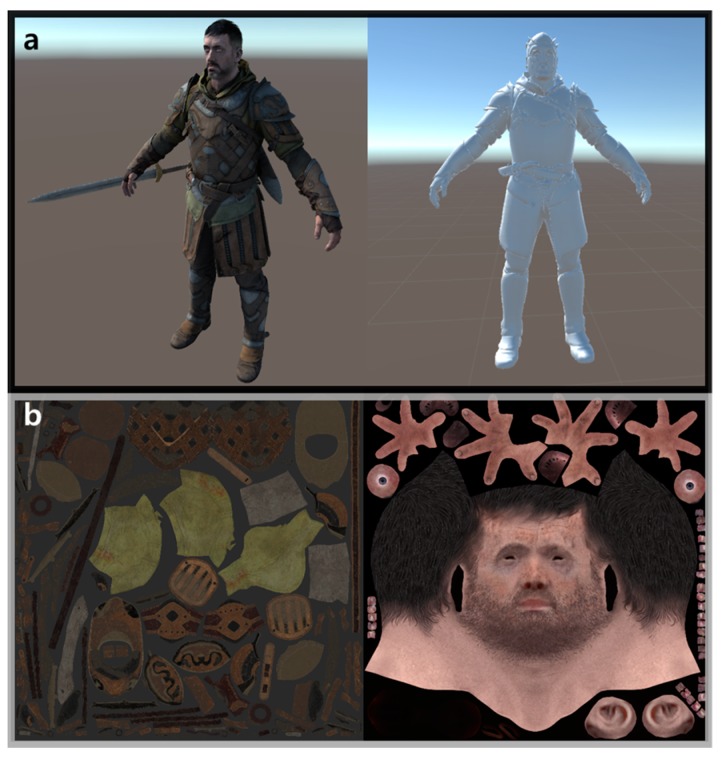
(**a**) A model with a high number of polygons used in the experiment and (**b**) textures of the model.

**Figure 17:**
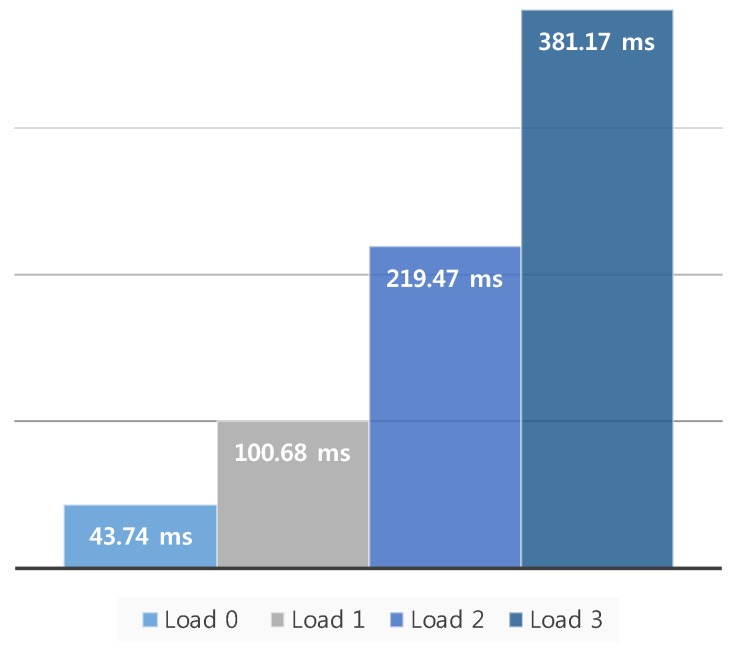
Change in the motion-to-photon latency according to the change in the graphics rendering load.

Second, the change in motion-to-photon latency was measured according to the change in the image rendering workload. Generally, the HMD system requires a high-performance GPU because it needs to generate 3D-rendered contents with high resolution and a variety of visual effects. However, the rendering process generates a latency, and this latency is changeable according to the workload for rendering an output image. For example, if an output image is complex in the same hardware resource, the latency would increase. Therefore, in the following experiments, the change in latency was measured when varying the rendering workload in the HMD. The proposed measurement system used a model with a high number of polygons as a basic unit in the Unity game engine to set the rendering workload, as shown in Figure 16a. This model is composed of many textures, as shown in Figure 16b. Generally, the specific indicator that can predict the workload is a vertex. A vertex in computer graphics is a data structure that describes certain attributes such as the position of a point in 2D or 3D space, at multiple points on a surface [20]. Table 4 shows the number of vertices according to the rendering workload change. The rendering workload was gradually increased by increasing the number of vertices. In this experiment, the workload stage was largely divided into four steps. Load 0 was the normal state, with no additional model for the workload in the virtual space. Load 1 used 16 models that had 9.1 million vertices in the virtual space. Load 2 used 32 models that had 20.5 million vertices. Load 3 used 48 models that had 34.2 million vertices. Figure 17 shows the latencies for the different workloads from load 0 to load 3, and the motion-to-photon latency increased up to 381.17 ms when increasing the rendering workload. This means that the proposed device can accurately measure latency changes due to workload changes.

## 6. Conclusions

This paper proposed a new measurement system that can accurately measure the motion-to-photon latency. The system, which uses high-accuracy DC motors and encoders with high resolution, could replicate the head movement, and it is possible to measure the rotation angles of the platform in realtime. In addition, the luminance change in the display could be detected using a photosensor. Specifically, the system is composed of a head position model-based rotary platform, pixel luminance change detector, and signal analysis and calculation modules. The proposed system can exactly measure the motion-to-photon latency. The experimental results showed that the latency was measured to be up to 47.05 ms. Additionally, the measured latency increased up to 381.17 ms when increasing the rendering workload in the HMD.

The future work will focus on how to measure the motion-to-photon latency in successive frames using this developed measurement instrument. Specifically, the total system including the graphic user interface will be developed for tracking and expressing the head orientation by changing the intensity level of the image displayed in a HMD device.

## Figures and Tables

**Table 1:** Comparison of conventional and proposed measurement methods.

Previous Methods	Measurement Equipment	Considerations ^1^	Measurement Coverage (Reference to Figure 1)
Choi & Seo [10]	Photosensor	ⓐ, ⓑ, ⓓ, ⓕ	②~⑦
Steed [11]	Video camera	ⓐ, ⓓ	④~⑦
Zhao [12]	Photosensor Rotary potentiometer	ⓑ, ⓔ, ⓕ	①~⑦
Giorgos & Leigh [13]	Photosensor Rotary motor	ⓑ, ⓒ, ⓓ	①~⑦ (Except ④)
Lincoln [7]	Photosensor Rotary encoder	ⓑ, ⓒ, ⓔ	①~⑦
Proposed method	Photosensor Rotary encoder	ⓑ, ⓒ, ⓔ, ⓕ	①~⑦

Considerations ^1^: ⓐsimple implementation, ⓑhigh sampling rate, ⓒphysical movement consideration, ⓓ use of PC monitor (mirroring), ⓔ use of display panel (direct method), and ⓕ used for latency measurement of various types of commercially available HMDs.

**Table 2:** Average latencies and standard deviations for different rotation angles (yaw rotation).

Conditions		Measured Time (ms)	
Max Rotation Angle (°)	Angular Velocity (°/s)	Average Latency	Standard Deviation
20	42.82	44.61	1.45
40		46.83	0.74
60		46.46	0.90

**Table 3:** Average latencies and standard deviations for different rotation angles (pitch rotation).

Conditions		Measured Time (ms)	
Max Rotation Angle (°)	Angular Velocity (°/s)	Average Latency	Standard Deviation
10	42.82	46.48	1.09
20		46.79	0.98
30		47.05	0.89

**Table 4:** The number of vertices for workload change.

	Workload			
	0	1	2	3
**# of models**	0	16	32	48
**Vertices**	148	9.1M	20.5M	34.2M
